# Influence of Centrifugation and Shaking on the Self-Assembly of Lysozyme Fibrils

**DOI:** 10.3390/biom12121746

**Published:** 2022-11-24

**Authors:** Marzena Krzek, Sander Stroobants, Pierre Gelin, Wim De Malsche, Dominique Maes

**Affiliations:** 1 Structural Biology Brussels, Vrije Universiteit Brussel, 1050 Brussels, Belgium; 2 μFlow Group, Department of Chemical Engineering, Vrije Universiteit Brussel, 1050 Brussels, Belgium

**Keywords:** fibrilization, protein self-assembly, mass transfer, centrifugation, shaking, hypergravity, oligomers

## Abstract

Protein self-assembly into fibrils and oligomers plays a key role in the etiology of degenerative diseases. Several pathways for this self-assembly process have been described and shown to result in different types and ratios of final assemblies, therewith defining the effective physiological response. Known factors that influence assembly pathways are chemical conditions and the presence or lack of agitation. However, in natural and industrial systems, proteins are exposed to a sequence of different and often complex mass transfers. In this paper, we compare the effect of two fundamentally different mass transfer processes on the fibrilization process. Aggregation-prone solutions of hen egg white lysozyme were subjected to predominantly non-advective mass transfer by employing centrifugation and to advective mass transport represented by orbital shaking. In both cases, fibrilization was triggered, while in quiescent only oligomers were formed. The fibrils obtained by shaking compared to fibrils obtained through centrifugation were shorter, thicker, and more rigid. They had rod-like protofibrils as building blocks and a significantly higher β-sheet content was observed. In contrast, fibrils from centrifugation were more flexible and braided. They consisted of intertwined filaments and had low β-sheet content at the expense of random coil. To the best of our knowledge, this is the first evidence of a fibrilization pathway selectivity, with the fibrilization route determined by the mass transfer and mixing configuration (shaking versus centrifugation). This selectivity can be potentially employed for directed protein fibrilization.

## 1. Introduction

Protein aggregation is the process that leads to the formation of protein assemblies such as “oligomers” or “amyloid fibrils”, typically arising from non-native intermolecular contacts (e.g., protein misfolding) [1]. In addition to aggregation, other pathways exist to form a plethora of soluble oligomers [2]. Several proteins are notorious to form amyloid fibrils that contribute to human pathologies, such as the Aβ-peptides in Alzheimer’s disease and α-synuclein in Parkinson’s disease or medin, a protein implicated in cardiovascular disease [3,4]. Intrinsically disordered proteins can contain aggregation-prone regions, which makes them more likely to form amyloid fibrils spontaneously (in vitro) compared to folded proteins. For the latter ones, it is imperative to expose the aggregation-prone region to the protein surface, and this is accomplished by destabilizing the protein conformation and partial unfolding. Apart from traditional approaches to provoke and influence fibril formation (change in pH, change in temperature, the addition of chemical denaturants [5,6], mutations [7,8]) some studies revealed that agitation stress such as stirring, and shaking can cause fibrilization [9]. At present, this poses an increasing challenge to many companies producing protein pharmaceuticals, because aggregates of therapeutic proteins can induce immune responses that render them useless and potentially even dangerous for pharmaceutical purposes [10]. Moreover, controlled self-assembly can also be a critical step in peptide drug formulation [11] or materials production [12,13,14].

**Figure 1:**
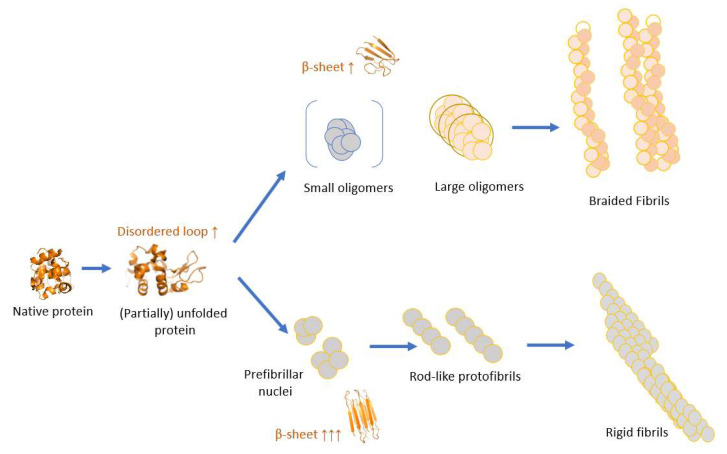
The fibrilization process for folded proteins starts with a partial unfolding step. There are two main pathways that protein fibrilization can follow; the top pathway shows the oligomeric pathway where a small structural reorganization occurs (typically enrichment with β-sheets), and protein molecules agglomerate into oligomers. Oligomers can associate into fibrils with a braided fibril morphology [15,16,17]. The bottom pathway shows the oligomeric-free assembly (also called nucleated polymerization [18]), where a more profound structural rearrangement occurs resulting in prefibrillar nuclei. In the elongation or growth phase, these nuclei are extended into an organized conformation with the cross β-structure (protofibril) whereas in the steady phase the protofibrils associate into mature fibrils. The scheme is based on [19,20,21].

There are two main fibrilization pathways, both initiated with a partial protein unfolding (Figure 1). The main factor for selecting a pathway is believed to be the critical oligomeric concentration [19], but also other chemical conditions seem to play an important role [17]. The choice of pathway does not only determine the final morphology of the fibrils but also their physiological response [22,23]. Along the oligomeric pathway, the protein molecules undergo reorganization, herewith primarily losing the α-helical content and forming oligomers (Figure 1 top part) which further rearrange and either associate into braided fibrils or remain in solution as off-pathways oligomers [24]. The oligomeric-free pathway starts with a more profound structural rearrangement and enrichment in the β-sheet content of the protein molecules that assemble into prefibrillar nuclei. In the elongation or growth phase, the nucleus is extended into a well-defined conformation containing a cross β-structure (protofibril), that associates into mature rigid fibrils. Fibrils assembled from oligomers have a very distinct morphology compared to rigid fibrils, they are braided (also called curvilinear), more curved, can display periodicity in their height along their length [25], and coexist with and between oligomers [26]. The two types of protein fibrils (Figure 1) are reported for a wide range of proteins [17] including Aβ peptide [27], lysozyme [28], and the prion protein [15]. The formation of one or the other form has been reported to be predetermined by several factors including chemical conditions e.g., pH [13,17], mutations [25], salt concentration [29], air–water interface [30], and presence of agitation [27,31,32,33].

The physical phenomena underlying the effect of mass transport on the fibrillization process are not fully understood and the literature on this topic is scarce. Mass transfer within liquid solutions can be divided into two main types (1) advection, which is the transport of the liquid as bulk motion, e.g., in a pressure-driven flow; (2) movement of solute(s) under the influence of an external force. The first type of mass transfer is broadly present in living organisms, for instance in pulsatile moves of cerebral fluids [34], cytosol in cells, or flow through the vessels. Additionally, it underlies almost every step of upscaled protein production and processing. Dunstan et al. reported that advective transport caused by shear triggers structural destabilization: very subtle structural disruptions were sufficient to alter the fibrilization kinetics or its mechanism [35]. The second type of mass transfer occurs in every process where altered accelerations are involved: at the roller coaster, during the launch of a space rocket, in an elevator, and in industrial processes using sedimentation and centrifugation. To our knowledge, the effect of an increased acceleration force on the fibrilization process has not been studied and described. At the same time, the literature on microgravity effects is scarce. Morphological [36] and kinetical alterations [37] were reported for samples fibrillating in microgravity. Interestingly, flows and gravitational forces are directional, and it was reported that the geometry of the applied forces plays a role in the destabilization of the protein [38].

In this paper, we discuss the effect of centrifugation, on the fibrilization process of the model protein hen egg white lysozyme and compare our results with “classical” shaking using a benchtop orbital shaker. We analyze the characteristics of the aggregates obtained in both regimes: their morphology, supramolecular structure, and surface hydrophobicity.

## 2. Materials and Methods

### 2.1. Chemicals and Solutions

All reagents used were of analytical grade and were obtained from Sigma-Aldrich or VWR. Lyophilized hen egg white lysozyme was purchased from Thermo Scientific. All the solutions were freshly prepared in MiliQ water and filtered with 0.2 μm PTFE syringe filters (Whatman) before use. The lysozyme concentration was measured with the Thermofisher Scientific NanoDrop One.

### 2.2. Fibrilization

Lysozyme was dialyzed against 50 mM sodium acetate buffer pH 4.5. The fibrillization solution containing 12, 20, or 40 mg/mL of lysozyme, and 15% of ethanol at pH 1.5 (adjusted with HCl) was prepared directly before the experiment. All solutions were filtered with 0.2 and 0.1 μm PTFE syringe filters (Whatman) before pH adjustment and were prepared with minimal agitation and no extra stirring or shaking. The Eppendorf tubes (see Supplementary information for detailed dimensions, Figure S1 and S2) were placed in triplicate in three different mass transport regimes for eight days at a constant temperature of 42 °C: (1) in an Eppendorf rack (referred to as 1 g in the rest of the text), (2) tabletop centrifuges (Microcentrifuge ^®^16 Beckman Coulter, Pasadena, CA, USA) running at 521 and 1646 rpms corresponding to 20 and 200 g acceleration, respectively, (referred to as 20 g and 200 g), (3) in an Eppendorf Thermomixer Comfort running at 750 rpm (referred to as shaking).

### 2.3. Dynamic Light Scattering (DLS)

Dynamic light scattering experiments were performed with a DynaPro^®^ NanoStar^®^ instrument (WYATT technology, Santa Barbara, CA, USA). Data analysis was performed with the DYNAMICS software package. The analyzed correlation curves were averages of ten measurements of 7 s.

### 2.4. Atomic Force Microscopy (AFM)

After fibrilization, 15 μL of each sample was diluted 20–100-fold into MiliQ water adjusted to pH 2 with HCl, deposited on freshly cleaved mica, and incubated for 20 min, 10 times rinsed with 100 μL of MiliQ water and dried with air. The dry mica discs were analyzed with a Nanoscope IIIa multimode atomic-force microscope (Veeco, Santa Barbara, CA, USA). The instrument operated in tapping mode with edged silicon tips (NCHV-A, Bruker). Images were acquired at 216 × 216 and 512 × 512 pixel resolution for areas of 2 μm × 2 μm and 12 μm × 12 μm, respectively. The raw image data were processed with the Nanoscope IIIa software. A first- and second-order flattening was applied. The height (profiles), cross sections, end-to-end distance, and lengths of the fibrils were obtained using FiberApp open-source software [39]. The rigidity of a fibril was determined by calculating its persistent length as described in [40]. From each sample, 17 representative fibrils were analyzed from 3 different images.

### 2.5. Fourier Transform Infrared Spectroscopy (FTIR)

Samples were filtered with a 0.1 μm filter (Ultrafree-MC Centrifugal Filter, Merck, Darmstadt, Germany, 0.1 μm PVDF). The retentate was washed on the filter with a 50 mM deuterated glycine buffer pD 1.5. Both fractions as well as the entire solution mixture were vacuum dried at 43 °C for 6 h and resuspended in 50 mM deuterated glycine buffer at pD 1.5. FTIR measurements were performed on the retentate, the permeate, and the total fraction immediately after dissolution or washing. The measurements were performed on a Cary 630 FTIR spectroscope (Agilent Technologies, Santa Clara, CA, USA). The laser power was set to a gain of 2000 and a wavelength range of 2000 to 900 cm^−1^ was scanned with a resolution of 0.25 cm^−1^. Every spectrum was collected as an average of ten scans. For the structural analysis, the amide I region (1700 to 1600 cm^−1^) was deconvoluted into Voigt peaks [41] at the maxima within the region 1616–1627 cm^−1^ for the parallel β-sheets, 1628–1634 cm^−1^ for antiparallel β-sheets, 1643–1647 cm^−1^ for unordered part, 1650–1657 cm^−1^ for α-helix and 1660–1675 cm^−1^ for turns and loops [42,43,44].

## 3. Results and Discussion

We investigated solutions of lysozyme at three different concentrations (12, 20, and 40 mg/mL) under the following destabilizing conditions: elevated temperature (42 °C), acidic (pH 1.5), with an ethanol concentration of 15%. The solutions were incubated for 8 days in three different mass transport conditions: centrifugation at 20 and 200× *g* acceleration (hypergravity conditions), shaking in the thermoshaker and ground gravity as a control (referred to as 1 g or quiescent). The set-up details and characterization of mass transfer are described in Supplementary Information (Section 1, 2 and 3, Figure S1–4). It is well known that highly acidic conditions can induce proteolysis of the protein [45]. We confirmed by running an SDS-PAGE under reducing conditions that the lysozyme molecules remain intact after shaking and centrifugation (Supplementary Information Section 5 and Figure S8). All the Eppendorf tubes were fully filled with no air bubbles in order to exclude a possible air–liquid interface effect.

### 3.1. Shaking and an Increased Gravitational Field Triggers Fibrilization

**Figure 2:**
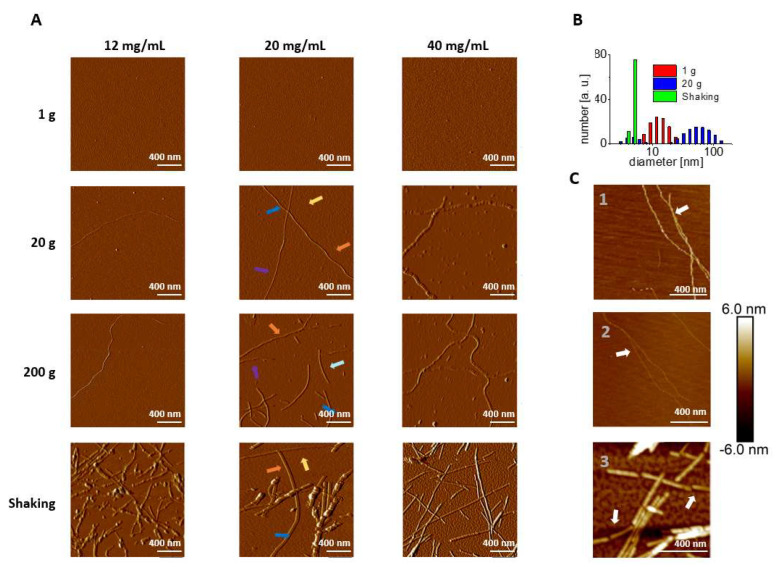
(**A**) Amplitude AFM images of lysozyme samples at pH 1.5 containing 15% ethanol at 12, 20, and 40 mg/mL after 8 days of incubation at 42 °C in 1 g, 20 g, 200 g, and shaking. The size of the images is 2 μm × 2 μm. The colorfed arrows indicate fibrils used in the morphology analysis. Images with a smaller magnification are given in Supplementary Information Figures S5 and S6. (**B**) Size distribution in the permeate from a 0.1 μm centrifugation filter of the entire fibrilization mixture in quiescent conditions, 20 g, and shaking determined by DLS. The fibrilization mixture contained 40 mg/mL of lysozyme at 42 °C in a solution at pH 1.5 with 15% ethanol. (**C**) Zoom-in of AFM height images of fibrillar aggregates from (1) centrifugation at 20× *g*; the arrow indicates lateral assembly by protofilaments interwinding; (2) centrifugation at 200× *g*; (3) shaking; the arrows indicate lateral assembly of separate, protofibril segments with a length of around 100–200 nm.

After eight days, fibrils were present in lysozyme solutions exposed to shaking and centrifugation, while no fibrils or protofibrillar formation was detected by AFM in samples left in quiescence (Figure 2A, Figures S5 and S6). In the samples held at 1 g, oligomeric globular formations were detected, which proves aggregation-prone conditions are not sufficient to initiate fibrilization within eight days. Note that fibrils were never observed at 1 g also for longer times (longer than 3 weeks).

To obtain an estimate of the amount of protein that was incorporated in the fibrils, the fibrilization mixture was filtered (cut off 0.1 μm) and the lysozyme concentration of the permeate was determined with UV–Vis absorption at 280 nm using an extinction coefficient of 2.64 mL mg^−1^ cm^−1^. The data revealed that 96 ± 1%, 31 ± 4%, and 42 ± 4% of the initial material was present for 40 mg/mL samples from 1 g, 20 g, and shaking, respectively. This indicates that most lysozyme molecules were not aggregated in the quiescent solution, as compared to more than 50% for the other conditions. Moreover, it was qualitatively observed that the number of fibrils obtained with shaking was larger than for the case when centrifugation was used. The high viscosity and the inhomogeneity of the sample prevented a reliable quantitative evaluation.

### 3.2. Influence of Mass Transfer Configuration on Fibril Properties

#### 3.2.1. The Height and Length of the Fibrils

**Figure 3:**
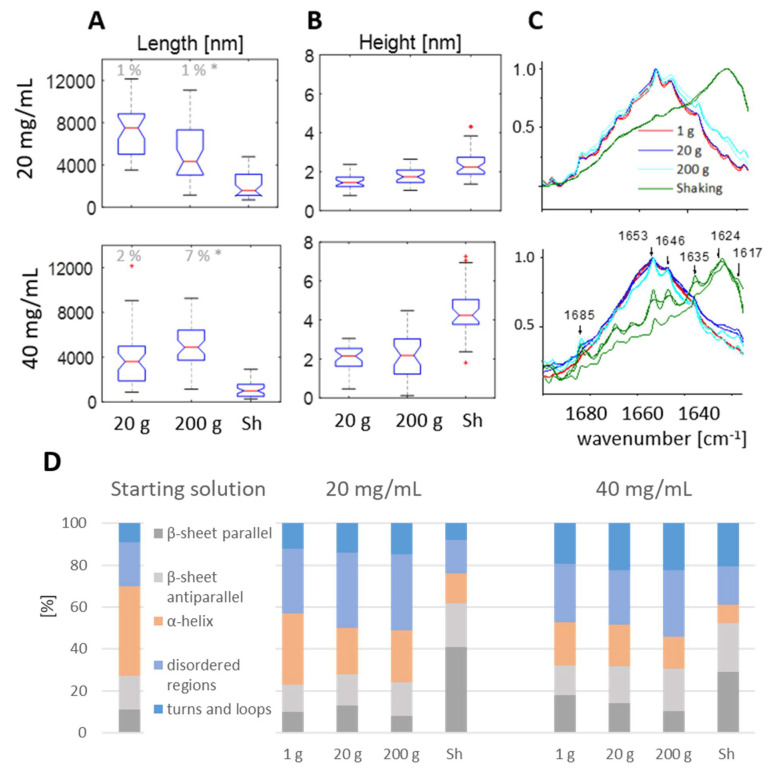
(**A**) Boxplots presenting the comparison of lengths and (**B**) heights of fibrils formed in the three studied agitation conditions (centrifugation at 20× *g*, 200× *g,* and shaking) for two lysozyme concentrations. Each boxplot summarizes 35 measurements. Red crosses indicate outliers, e.g., data points larger or smaller than 1.5 times the interquartile range below or above the first quartile or third quartile, respectively. The percentage of fibrils on the image that is not fully fitting the image but has a visible length longer than 6 μm is indicated on top of the boxplot; (**C**) overlap of normalized IR spectra for each condition, (**D**) percentage of secondary structures in different fibrilization mixtures for two lysozyme concentrations compared to the initial solution immediately after adaptation to 2 at 42 °C. Lysozyme samples were prepared at pH 1.5 with 15% EtOH and studied after 2 weeks of appropriate agitation at 42 °C.

The height and length of the fibrils were determined from AFM images with sizes 2 μm × 2 μm (Figure 2A) and 12 μm × 12 μm (Figure S5), respectively, for samples with 20 and 40 mg of lysozyme. Boxplots (Figure 3A) display the comparison of those two features between the fibrils formed within the studied mass transfer regimes. It is clear that the fibrils formed at 20 g and 200 g are longer and thinner than the ones obtained by shaking. Scheffé’s test (α = 0.01) revealed that the height differences were always significant between the samples that had been shaken and the samples from the remaining conditions. Additionally, the fibrils formed in 20 g were always significantly longer than the fibrils developed through shaking. For samples containing 40 mg/mL of lysozyme, the difference between the fibrils from 200 g and those that had been shaken was also valid. However, this dependency is not statistically significant for samples containing 20 mg/mL.

Note that the presented length distribution has limitations because fibrils that were partially outside the field of view were not considered. Nevertheless, the percentage of fibrils not fully fitting within the field of view, having a measurable length longer than 6 μm (half of the maximal measurable length), indicated on top of the boxplot is lower than 7%. For fibrils grown through shaking, all fibrils had a length far below 6 μm. Samples with 12 mg/mL of lysozyme had more than 30% of the fibrils outside the field of view, therefore we did not perform analysis on those samples.

The observed length differences indicate a higher elongation rate for fibrils formed in centrifugal acceleration as compared to fibrils from shaking. Note that this result might be influenced by fibril breakage due to extensive orbital shaking. Fibrils obtained in shaking are significantly thicker and are indicative of a higher-order assembly. At 20 g solutions containing 20 mg/mL have longer fibrils as compared to solutions at 40 mg/mL. This can be explained by the fact that at lower concentrations less fibrils nucleate, the growth is slower and within the same timescale they are thinner. The greater thickness of fibrils from 40 mg/mL compared to the fibrils from 20 mg/mL is in accordance with the suggested mechanism.

Altogether, the observed length and height differences seem to indicate that shaking favors the lateral association of rods while centrifugation promotes growth at the fibril tip.

#### 3.2.2. The Rigidity of the Fibrils

Fibrils formed in shaking are straight and do not bend. In contrast, fibrils formed in hypergravity turn and bend frequently within the same fibril-length scale (Figure 2A). For fibrils obtained at 40 mg/mL, we determined the persistence length as 52, 47, and 215 ± 1 nm for 20, 200 g, and shaking, respectively. We can conclude that fibrils formed in shaking are more rigid than fibrils formed in the centrifuge as their persistence length is around 4–5 times longer. The 17% difference between fibrils from 20 g and 200 g is marginal. The persistence length of fibrils from 20 g and 200 g is in the similar range as values reported for “worm like” fibrils, the analog of braided fibrils, of bovine albumin, or β-lactoglobulin [40]. In the literature, persistence lengths above 1 μm have been reported for rigid fibrils from shaking [46]. Note that for entropic reasons longer fibrils have a tendency to bend.

#### 3.2.3. Fibril Morphology at the Protofilaments Level

AFM images (Figure 2C) show significant differences in fibril morphology and filament assembly between samples exposed to different mass transfers. To quantify these differences the height profile of fibrils longer than 500 nm not crossing other particles or fibrils was performed from high-resolution AFM images.

**Figure 4:**
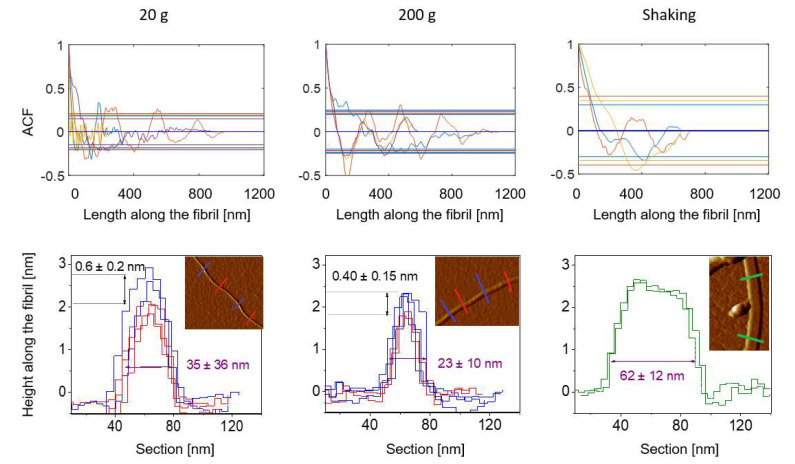
Morphological features of fibrils formed at different agitation modes for 20 mg/mL. Upper row—autocorrelation plots of fibril height profile. The color of the line corresponds to the arrows on the AFM image in Figure 2; horizontal lines represent 90% confidence thresholds; bottom row—cross-section height profile of fibrils. Red lines and blue lines correspond to the highest and lowest point of a periodic fibril, respectively. Green lines correspond to the cross-section of non-periodic fibrils. The graphs inserts are AFM images of the analyzed fibrils. The marked cross-sections have the same color as their plots on the corresponding graphs. Lysozyme samples were prepared at pH 1.5 with 15% EtOH and studied after 2 weeks of appropriate agitation at 42 °C.

Autocorrelation analysis shows that periodic fibrils with a periodicity around 310 ± 50 nm formed in 20 and 200 g for a lysozyme concentration of 20 mg/mL (Figure 4). In addition to that, AFM images provided solid data related to protofibrillar organization. The cross-sectional height profiles of fibrils formed in hypergravity were triangular with a height difference of around 0.5 nm between their highest and lowest points. This indicates the intertwining of long filaments [47], which was also observed directly on the AFM images (Figure 2C1-C2)). At the same time, fibrils obtained by shaking were flat, and their cross-section profile was closer to rectangular indicating no intertwining in the lateral association. Edge-to-edge protofibrillar assembly was detected (Figure 2C3) [48]. The protofibrils were rod-like with lengths between 90–190 nm and thickness between 3 to 6 nm. From the AFM images, we could see their end-to-end assembly resulting in a segmented fibril elongation (Figure 2C2). In contrast, single filaments from samples formed in hypergravity have thicknesses around 1.2 nm. It indicates that periodic fibrils are formed from homogeneous building blocks, e.g., long filaments formed from monomers of lysozyme (Figure 2C).

Full morphological homogeneity was found within the individual fibrils formed in shaking (100% flat fibrils), while for samples from 20 g homogeneity was less significant (80% periodic fibrils), and even less for fibrils from 200 g (60% periodic fibrils).

Taken together, our results show that the type of mass transfer, e.g., centrifugation and shaking, can determine different mechanisms of protofilaments assembly resulting in a different prefibrillar and consequently different fibrillar structures.

Few reports, describing fibrils mostly from Aβ peptide, show that non-rigid fibrils are formed in quiescent conditions while the rigid ones in agitation [27,32,49,50]. The authors postulate that a plausible mechanism for this effect is the enhanced water–air interface in agitation [27] and a change in selectivity or stabilization of different intermediate-formed symmetries during the nucleation and further assembly [50]. It is well known that the air–water interface can have a significant effect [14,30,51]. Since our Eppendorf tubes were fully filled, the air–liquid interface was reduced to a minimum. The differences in the submolecular organization are addressed below.

### 3.3. Submolecular Structural Features of the Lysozyme Mixtures

To get more insight into the different fibrillization pathways for hypergravity and shaking, the permeate for the presence of oligomers after filtering (0.1 µm filter) the fibrilization mixture was checked. The DLS data revealed that samples from shaking solutes with diameters up to 5 nm are dominant (Figure 2B). These solutes can be ascribed as monomers and very small agglomerates (up to three molecules), consistent with the oligomeric free pathway.

For the 20 g permeate sample at least two components of oligomeric assemblies are present, one within the size range of 2.5–8.5 nm and the second one between 20 and 100 nm (100 nm is the cut-off of the filter). This is consistent with our AFM results wherein protofibrils were detected next to oligomers. For the quiescent samples, one population of oligomers with sizes ranging from 7 to 20 nm was observed, indicating that oligomer growth and elongation were not promoted in this condition.

We performed an FTIR analysis to get more insight into the submolecular structural features of the mixtures. The results were compared with freshly dissolved lysozyme in pD 1.5 and 15% ethanol and are shown in Figure 3C. For both concentrations, the α-helical content decreased in all the fibrillization mixtures. The spectra from shaking are strikingly different from the others in the amide I region represented by distinct peaks between 1616 and 1636 cm^−1^, the β-sheets region. A deconvolution analysis shows that the percentage of β-sheet, as well as parallel and anti-parallel, is significantly higher than in other samples, and the random coil is lower (Figure 3D). In addition, the retentate after filtration on a 0.1 µm filter, consisting of fibrils and large oligomers, contains parallel β-sheet organization associated with the amyloid structure in contrast to all the other retentates and all remaining permeates—also from shaking (Supplementary Information Section 4 and Figure S7) [52]. The spectra of the lysozyme mixture from 1 g, and centrifugation are similar to one another within the sample and within fractions features. They have increased random coil and antiparallel β-sheet content as compared to the initial solution. All their fractions have antiparallel β-sheets, represented by a peak around 1684 cm^−1^ ascribed to pro-fibrillar oligomers [53,54]. This shows that for these samples, the structural features of single molecules and their assemblies within larger aggregates are preserved, which demonstrates similarity in their building blocks with reasonable random coil content in all of them.

Altogether, this indicates that in contrast to fibrils formed in centrifugation, orbital shaking causes unique additional structural arrangements at the single molecular or very early assembly level which resulted in rigid fibrils with no detectable antiparallel features. This is in line with other reports describing rigid and braided fibrils [33]. Moreover, ThT binding affinity experiments show that the solution from shaking has a significantly higher affinity for this hydrophobic dye than the mixture from centrifugation, proving that the chemical specificity of the surface of the aggregates is different and confirming that the obtained fibrils display a higher degree of β-structure. At the same time, the close structural similarity of the quiescent and centrifuge samples indicates that non-prefibrillar oligomers develop in both solution types. The fact that at 20 g and 200 g fibrils are formed in contrast to quiescent conditions might be due to an alignment of the building blocks during sedimentation at higher g-levels.

## 4. Conclusions

Mass transfer is broadly present in living organisms and important for their correct function. Additionally, it underlies almost every step of upscaled protein production and processing. In this paper, we demonstrated that different mass transport configurations, in addition to chemical factors, can have an influence on the self-assembly pathway of aggregation-prone protein solutions. Although it is generally known that agitation and shear stress can trigger protein fibrillization we found that different assembly pathways were chosen for centrifugation (under mainly nonadvective mass transport) and shaking (under advective mass transport) resulting in different fibril morphology. Only rigid fibrils were formed along the oligomeric free pathway through orbital shaking at 750 rpm. In contrast, predominantly braided fibrils were formed along the oligomeric pathway under extended gravitational accelerations. No fibrils were obtained in quiescent solutions. AFM imaging showed that fibrils obtained by shaking were shorter, thicker, and more rigid than fibrils obtained with centrifugation. Based on FTIR data, we concluded that shaking conditions cause destabilization of the lysozyme molecule, initiating an oligomeric-free pathway of fibril formation, while destabilization by centrifugation up to 200× *g* is less pronounced and leads to an oligomeric pathway for most fibrils. A possible explanation might be that local flow, caused by shaking, exerts a significant shear on the lysozyme molecules (see Supplementary Information Section 3 for a detailed description of the agitation modes). Furthermore, the two observed types of fibrils have been proven to differ in their supramolecular structural organization. In contrast, the fibrils and oligomers obtained in 20 g and 200 g have the same structural characteristics as the oligomers from quiescent conditions.

This might indicate that the concerted centrifugal oligomer/(pre-)fibril motion in the centrifuge leads to an alignment of the oligomers initiating the formation of braided fibrils.

The results were consistent for different sizes and shapes of the tubes. Moreover, we confirmed that the lysozyme molecules remain intact in the solutions for all conditions and that the air–liquid interface has no effect.

It is unclear whether the oligomeric free and oligomeric pathways are unique and deterministic. Future experiments will address this matter.

Altogether, our results suggest that mass transport can be used as a tool to direct and modulate protein aggregation. Moreover, crucial steps in protein production like flows, sedimentation, and centrifugation seem to have a large impact on the protein self-assembly process.

## Data Availability

Not applicable.

## Supplementary Materials

The following supporting information can be downloaded at: https://www.mdpi.com/article/10.3390/biom12121746/s1, Section 1: Experimental set-up; Section 2: Characterization of the agitation modes; Section 3: Mass transfer characterization [55]; Section 4: Supramolecular structure of the fibril sample fractions - FTIR study; Section 5: Stability of lysozyme under employed conditions; Figure S1: Tubes used for studying fibrilization; Figure S2: Graphical representation of the orientation of the tube for (A) centrifugation, (B) shaking; Figure S3: (A) An Eppendorf tube with geometry B exposed to rotational movements during centrifugation and shaking; (B) contribution of inertial shear component to centrifugal force for the Eppendorf horizontal cross-sections; Figure S4: Eppendorf tubes with a solution of ThT and isopropanol: (A) initial solution, (B) solution after shaking by hand for 10 seconds, (C) solution after 1 hour in the thermoshaker and (D) solution after 1 hour in the centrifuge at 750 rpm corresponding to 20 g; Figure S5: AFM height images of lysozyme samples at pH 1.5 containing 15 % ethanol at 40 mg/mL after 8 days of incubation at 42 °C in 1g, 20g, 200g and shaking. Comparison of the different tube geometry at different magnifications; Figure S6: AFM height images of lysozyme samples at pH 1.5 containing 15 % ethanol at 12, 20 and 40 mg/mL after 8 days of incubation at 42 °C in 1g, 20g, 200g and shaking in geometry B; Figure S7: FTIR spectra of fractions after filtration with a 0.1 μm filter isolated from a sample incubated in different mass transfer regimes containing 40 mg/mL of lysozyme after fibrilization at 42°C in solution at pH 1.5 containing 15% ethanol; Figure S8: SDS-PAGE gel in denaturing conditions of the lysozyme solution (15 % ethanol at pH 1.5 incubated for 8 days in at 40 degrees): (1) sample form shaking, (2) sample from 20 g centrifugation and (3) quiescence.
