# Supplementary material for: Influence of Centrifugation and Shaking on the Self-Assembly of Lysozyme Fibrils
Source: Biomolecules. 2022 Nov 24;12(12):1746. doi: 10.3390/biom12121746 (PMC9775142; doi:10.3390/biom12121746)
Supplement: Supplementary file 1 [file biomolecules-12-01746-s001.zip › biomolecules-2022314-supplementary.pdf]

Influence of different mass transfers on the self-assembly pathway of lysozyme fibrils

M. Krzek[a,b], S. Stroobants[a], P. Gelin[c], W. De Malsche[c], D. Maes[a]

[a]Structural Biology Brussels, Vrije Universiteit Brussel, 1050 Brussels, Belgium
[b]Łukasiewicz Research Network—Industrial Chemistry Institute, 8 Rydygiera, 01-793 Warsaw, Poland
[c]µFlow group, Department of Chemical Engineering, Vrije Universiteit Brussel, 1050 Brussels, Belgium

1. Experimental set-up

We performed fibrilization experiments at different agitation modes orbital shaking, centrifugation with 20, 200 g and quiescent conditions in three different tube geometries (Figure S1) and at three different concentrations 12, 20, 40 mg/mL. Since all tube were fully filled with the fluid, the air-water interface factor has been excluded from this study and we can assume that the fibrilization solution was only in contact with the Eppendorf tube walls (hydrophobic polypropylene). Nevertheless, we also performed an experiment with half-filled tubes and we obtained the same results.

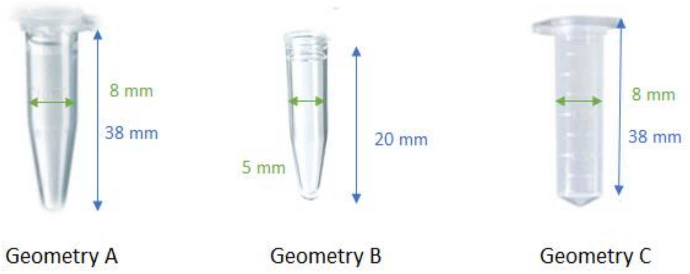

Figure S1. Tubes used for studying fibrilization. From the left: 1.5 mL Eppendorf tube (geometry A), PCR Eppendorf (geometry B), and 2mL Eppendorf tube (geometry C).

The AFM images show similar results for all geometries (Figure S5) and the different concentrations in geometry B (Figure S6) at 2 different magnifications. The thorough analysis reported in the paper was performed in geometry B.

2. Characterization of the agitation modes

We employed three different types of mass transfer: (1) quiescence (no agitation), (2) centrifugation (at 20 and 200 g acceleration), and (3) shaking in a tabletop thermoshaker. In the thermoshaker the sample is subjected to orbital movements as in the centrifuge but with a smaller radius: 1.5 mm versus 65 mm. Moreover, the orientation of the tubes upon orbiting remains unaltered for an external observer (Figure S2). In contrast in the centrifuge the tube rotates around its' axis.

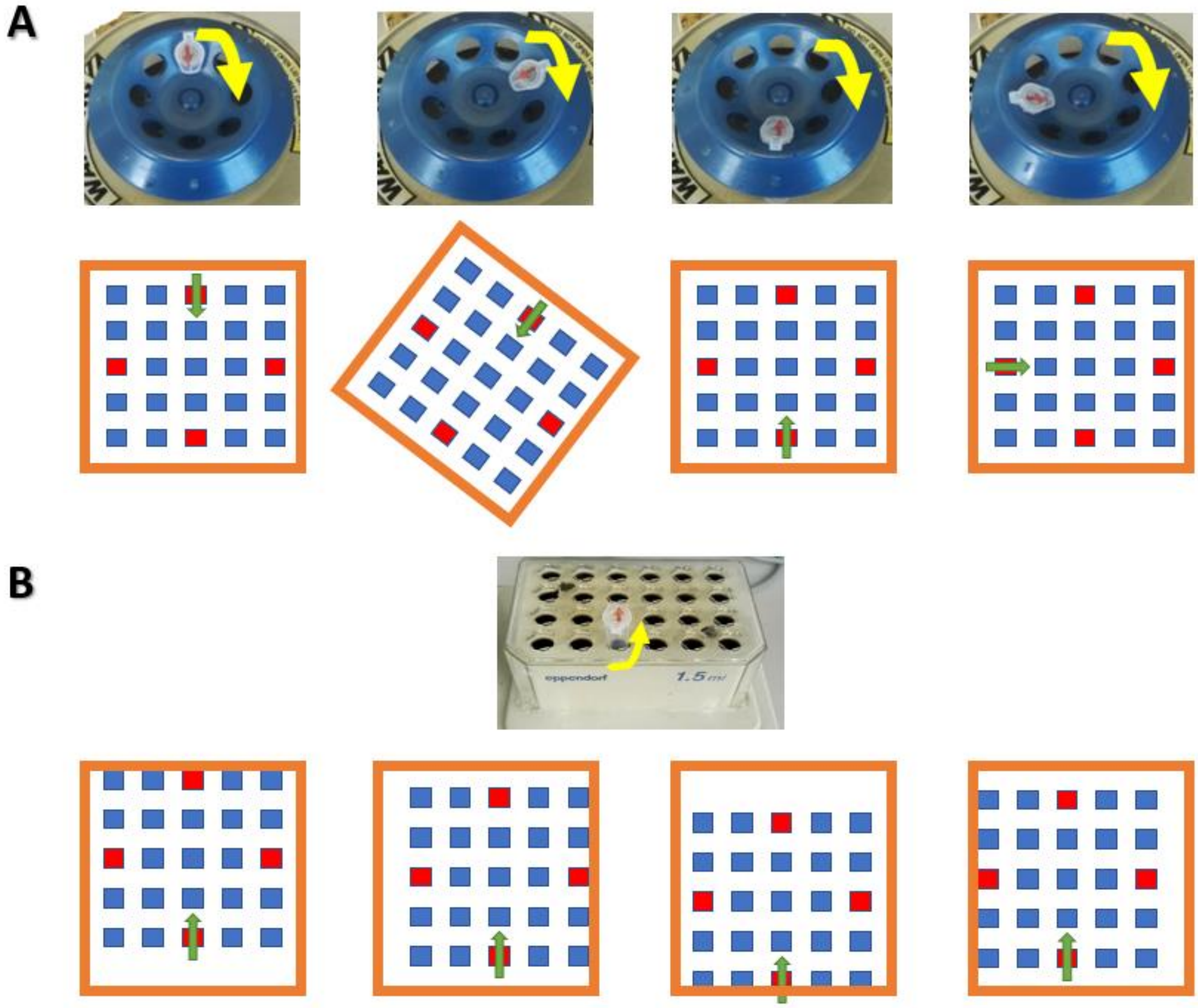

Figure S2. Graphical representation of the orientation of the tube for (***A***) *centrifugation,* (***B***) *shaking. In the thermoshaker the tube doesn't rotate along its axis.*

3. Mass transfer characterization

3a. Inertial shear

The centrifugal forces are not the same at every position in the sample as well in the centrifuge as in the thermoshaker. The magnitude of the centrifugal force depends on the distance from the rotation center and its direction is radial. Hence, for most positions in the sample the centrifugal force has a component perpendicular to the average centrifugal force exerted on the tube. The latter is responsible for internal shear as described in [55]. The ratio of the magnitude of this inertial shear component to the component along the average centrifugal force is shown in Figure S3. It is clear that this ratio is on average much larger in the thermoshaker as compared to centrifugation. This is a consequence of the smaller radius of rotation.

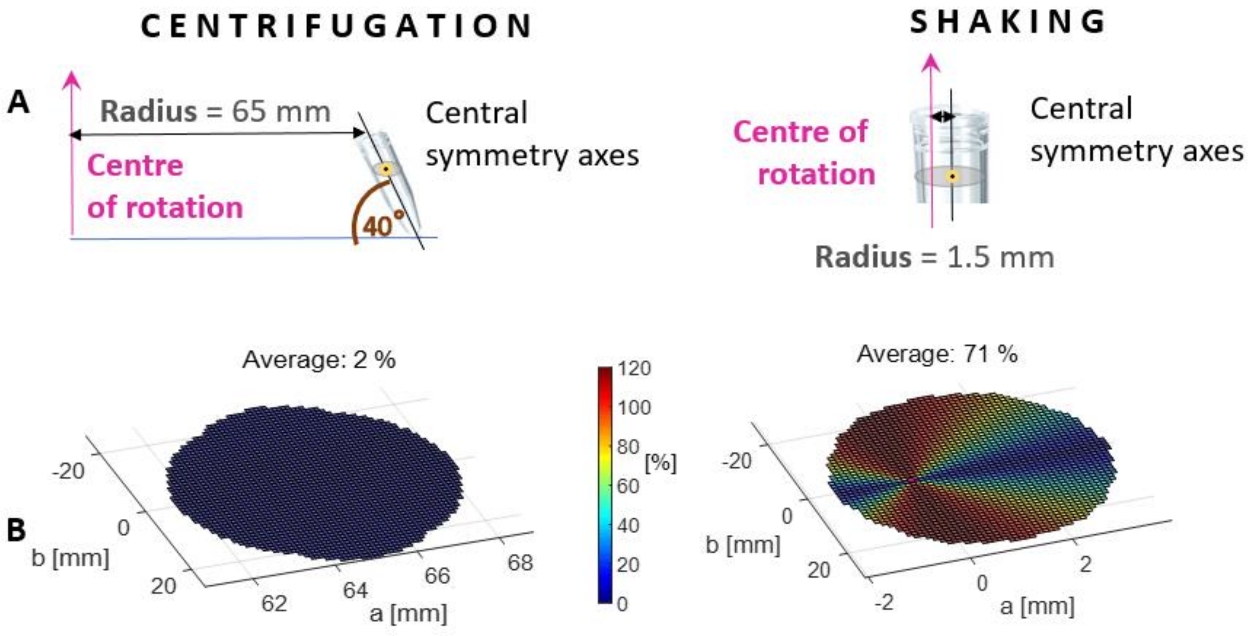

Figure S3. (*A*) *An Eppendorf tube with geometry B exposed to rotational movements during centrifugation and shaking; the axis of rotation is represented by a pink arrow. Grey zones in the Eppendorf tubes represent horizontal cross-sections;* (*B*) *contribution of inertial shear component to centrifugal force for the Eppendorf horizontal cross-sections (based on [1]).* The or*igin of the axis frame is at the center of rotation.*

3b. Macroscopic mass transfer models comparison

In order to visualize and compare the mixing under the different types of mass transfer we performed a simple experiment. We prepared 3 Eppendorf tubes with an aquatic solution (blue color from Coomassie blue dye) and isopropanol (yellow color from ThT) as pictured in Figure S6. The solutions had a clear interface, which is marked with a line on the tube wall (Figure S4 A). When shaking a tube by hand we observed that already after a few seconds both components mix and the interface disappears completely (Figure S4 B). Similarly in a tube placed in the thermoshaker for 1 hour both components are nearly completely mixed (Figure S4 C). In contrast, for a tube exposed to 1 hour of centrifugation at 750 rpm corresponding to 20 g the interface remained nearly undisturbed (Figure S4 D). This experiment clearly indicates that the mixing along the axis of the tube is more pronounced in the thermoshaker than in the centrifuge. Note that in this case the centrifugal force is perpendicular to the axis of the tube.

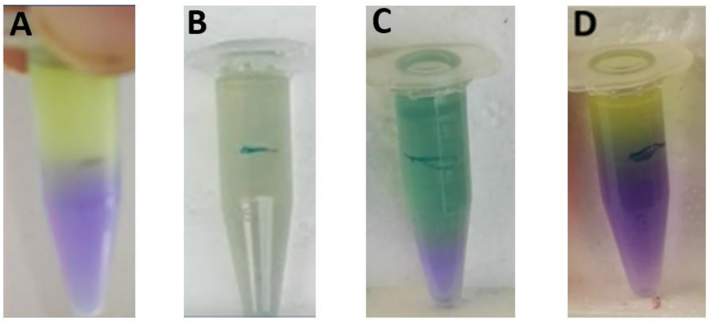

Figure S4. Eppendorf tubes with a solution of ThT (blue color) and isopropanol (yellow color): (*A*) *initial solution,* (*B*) *solution after shaking by hand for 10 seconds,* (*C*) *solution after 1 hour in the thermoshaker and* (*D*) *solution after 1 hour in the centrifuge at 750 rpm corresponding to 20 g.*

4. Fibrils formed under different conditions

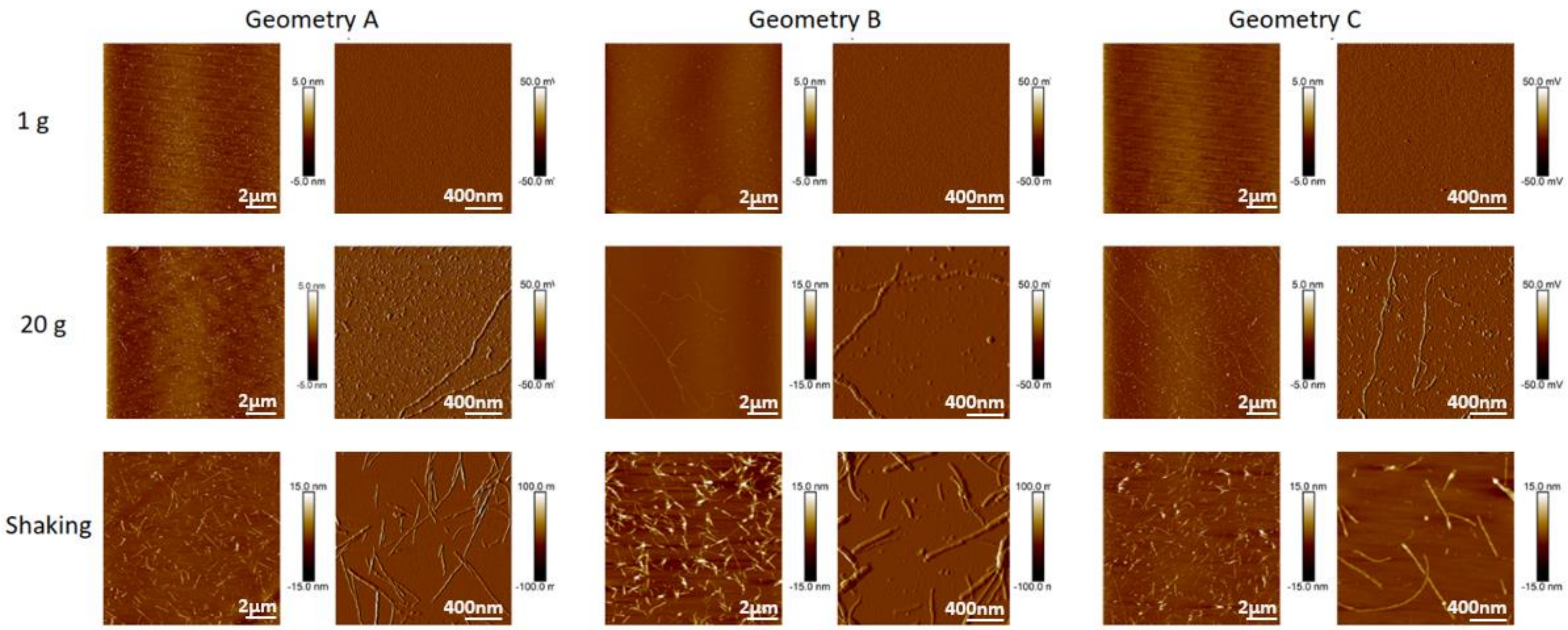

Figure S5. AFM height images of lysozyme samples at pH 1.5 containing 15 % ethanol at 40 mg/mL after 8 days of incubation at 42 °C in 1g, 20g, 200g and shaking. Comparison of the different tube geometry at different magnifications.

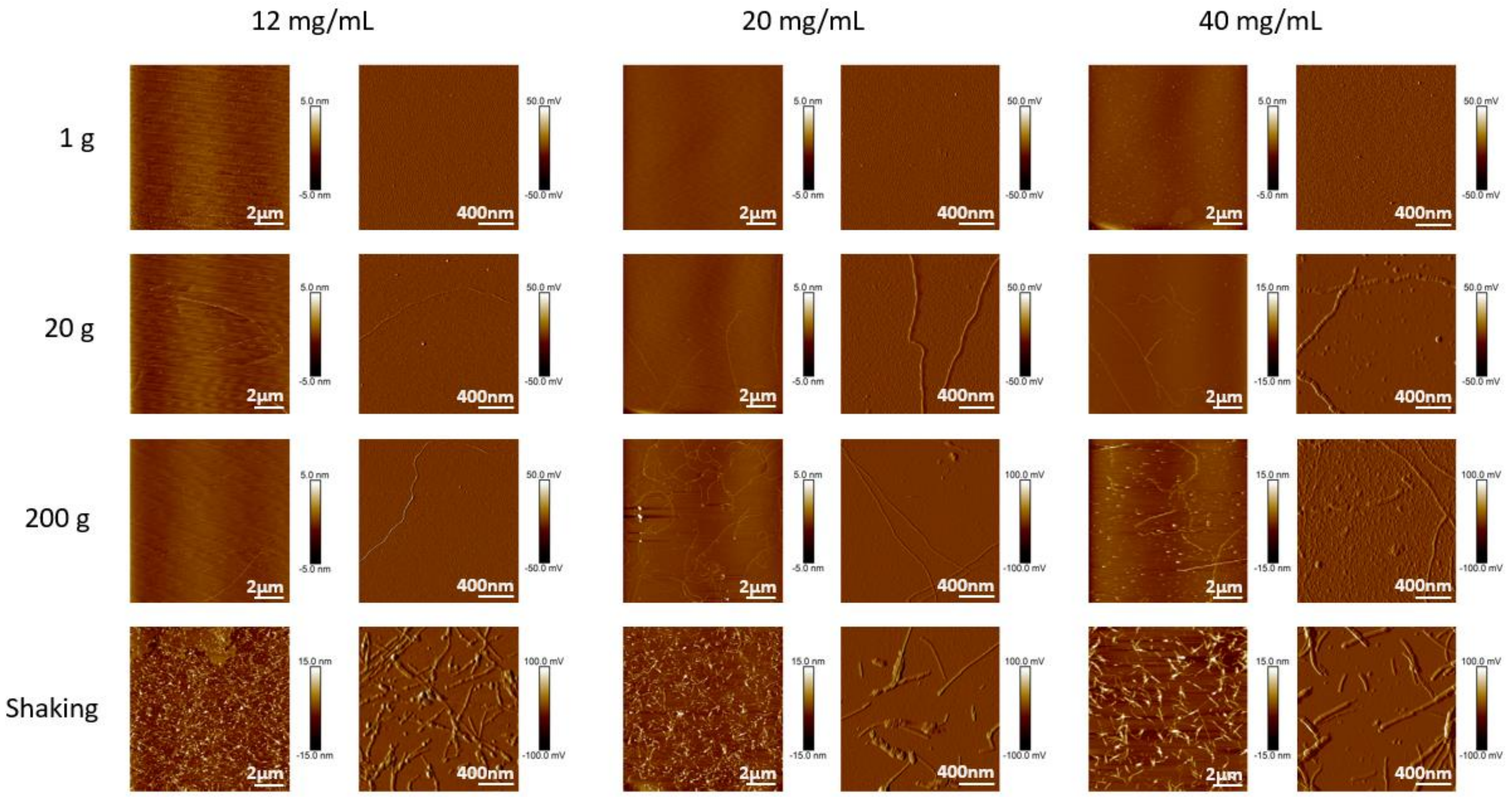

Figure S6. AFM images of lysozyme samples at pH 1.5 containing 15 % ethanol at 12, 20 and 40 mg/mL after 8 days of incubation at 42 °C in 1g, 20g, 200g and shaking in geometry B. For each concentration the column on the left represents height images and the column to the right amplitude images.

4. Supramolecular structure of the fibrils samples fractions – FTIR study

Lysozyme samples after eight days exposure to 42 °C with centrifugation at 20 g, 750 rpm shaking or incubation in quiescence were filtered (filter with 0,1 µm cut off membrane). Permeates and the flow throughs were studied separately by FTIR as described in materials and methods section. Results are presented below (Figure S7)

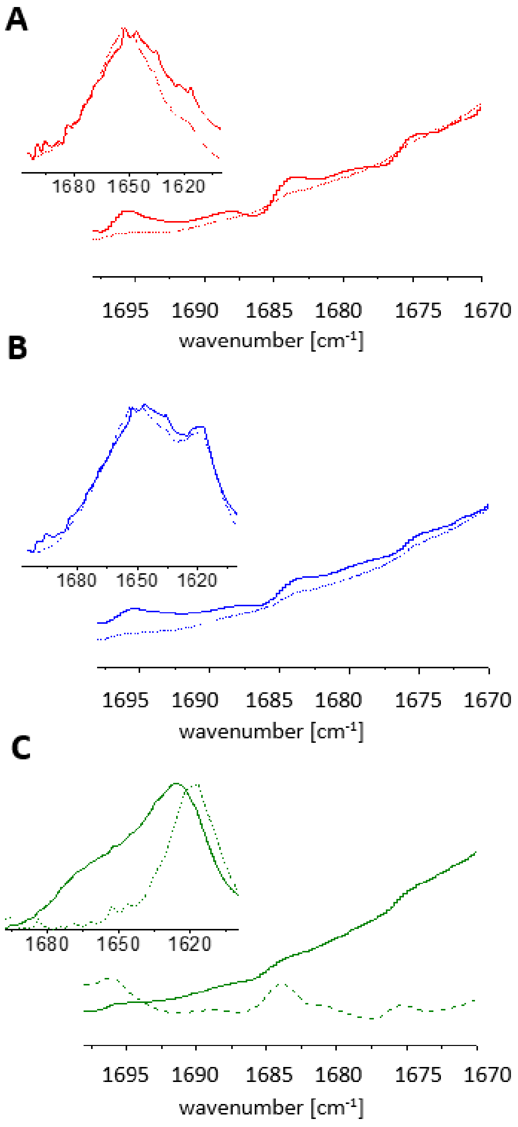

Figure S7. FTIR spectra of fractions after filtration with a 0.1 µm filter isolated from a sample incubated in different mass transfer regimes containing 40 mg/mL of lysozyme after fibrilization at 42°C in solution at pH 1.5 containing 15% ethanol. (A) red plots – 1 g; (B) blue plots 20 g; (C) green plots – shaking (C). Dotted line: fraction < 0.1 µm, full line – fraction > 0.1 µm.

5. Stability of lysozyme under employed conditions

Samples of lysozyme (40 mg/mL, 15 % ethanol at pH 1.5 incubated at 42 °C) were subjected to an SDS-PAGE analysis after preparation of the solution and after 8 days of fibrilization in the two different mass transfer regimes (thermoshaker and centrifugation at 20 g).

The SDS-reducing polyacrylamide gel electrophoresis was performed in a Tris–tricine buffer solution on 15 % gel using a BioRad setup. The loading buffer contained final concentration of 5 % of β-mercaptoethanol. Coomassie. Brilliant Blue G250 dye was used for staining.

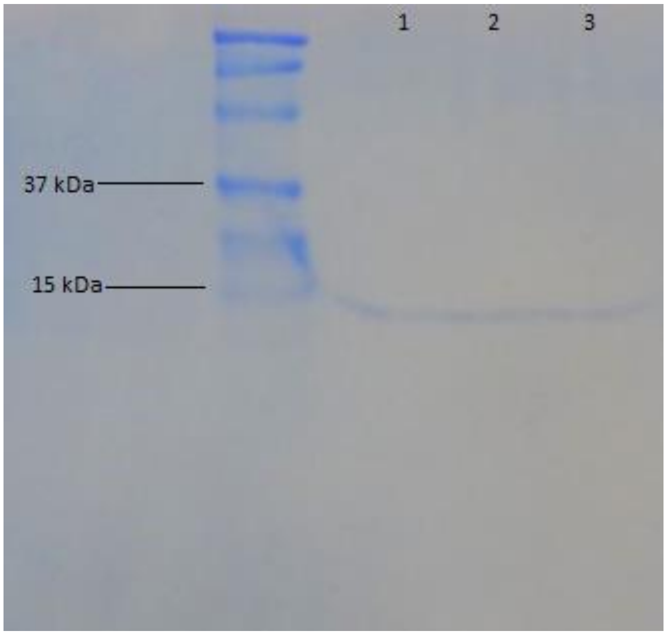

Figure S8. SDS-PAGE gel in denaturating conditions of the lysozyme solution (15 % ethanol at pH 1.5 incubated for 8 days in at 40 degrees): (1) sample form shaking, (2) sample from 20 g centrifugation *and* (*3) quiescence*

The results of the SDS PAGE analysis shows that the protein under conditions employed in this study remains intact: no protein degradation was detected (Figure S8).

References

55. van Loon, J.J.; Folgering, E.H.; Bouten, C.V.; Veldhuijzen, J.P.; Smit, T.H. Inertial shear forces and the use of centrifuges in gravity research. What is the proper control? *J. Biomech. Eng.* **2003**, *125*, 342–346.
